# *In situ* XPS data for the uranyl-modified oxides under visible light

**DOI:** 10.1016/j.dib.2018.06.121

**Published:** 2018-07-05

**Authors:** T.N. Filippov, N.S. Kovalevskiy, M.I. Solovyeva, I.A. Chetyrin, I.P. Prosvirin, M.N. Lyulyukin, D.S. Selishchev, D.V. Kozlov

**Affiliations:** Boreskov Institute of Catalysis, Lavrentieva 5, Novosibirsk 630090, Russian Federation

## Abstract

The silica, alumina, ceria, and titania supports were modified with uranyl ions (5 wt%) and investigated using X-ray photoelectron spectroscopy. The data show the U4f photoelectron spectra and charge state of uranium for uranyl ions deposited on different supports. The additional *in situ* XPS experiments with simultaneous irradiation of the sample using a 450 nm light-emitting diode were performed, and the XPS spectra, revealing a partial reduction of uranium under visible irradiation, are presented. The data show the effect of support material on the chemical states of uranium and oxygen on the surface of uranyl-modified oxides under visible light.

**Specifications Table**

**Table:** 

Subject area	Surface chemistry
More specific subject area	Photochemistry
Type of data	Figure, table
How data was acquired	XPS analysis: SPECS (Germany) spectrometer equipped with hemispherical PHOIBOS-150-MCD-9 analyzer and FOCUS-500 (AlK_α_ radiation, *hν* = 1486.74 eV, 200 W radiation power) monochromator
Data format	Analyzed
Experimental factors	Uranyl-modified oxides (5 wt%) were prepared via the impregnation of a support with UO_2_(NO_3_)_2_ aqueous solution followed by drying at 100 °C and grinding using an agate mortar and pestle. The sample preparation for XPS analysis was performed under room irradiation from luminescent lamps
Experimental features	XPS experiments were performed in *in situ* mode when the sample in a spectrometer chamber was simultaneously irradiated using a high-power light-emitting diode (LED) with a maximum at 450 nm. The survey spectra were taken at analyzer pass energy of 50 eV. The detailed U4f and O1s spectral regions were registered at 20 eV
Data source location	Boreskov Institute of Catalysis, Lavrentieva 5, Novosibirsk 630090, Russian Federation
Data accessibility	Data are accessible within this article
Related research article	T.N. Filippov, D.A. Svintsitskiy, I.A. Chetyrin, I.P. Prosvirin, D.S. Selishchev, D. V Kozlov, Photocatalytic and photochemical processes on the surface of uranyl-modified oxides: An in situ XPS study, Appl. Catal. A, Gen. 558 (2018) 81–90. doi:10.1016/j.apcata.2018.03.015

**Value of the data**

• Data show the photoelectron U4f and O1s spectral regions for different oxides supported with uranyl ions.
• New data about the uranium transformations on the surface of silica, alumina, ceria, and titania under the visible light are obtained using *in situ* XPS technique.
• Data show the effect of support material on the changing of uranium charge state under long-term visible irradiation.
• Data are useful for further studies on the elucidation of mechanisms for photochemical and photocatalytic reactions on the surface of uranyl-modified oxides.
• *In situ* XPS technique has great promise for the investigation of photochemical processes on the surface of different materials.

## 1 Data

**Fig. 1:**
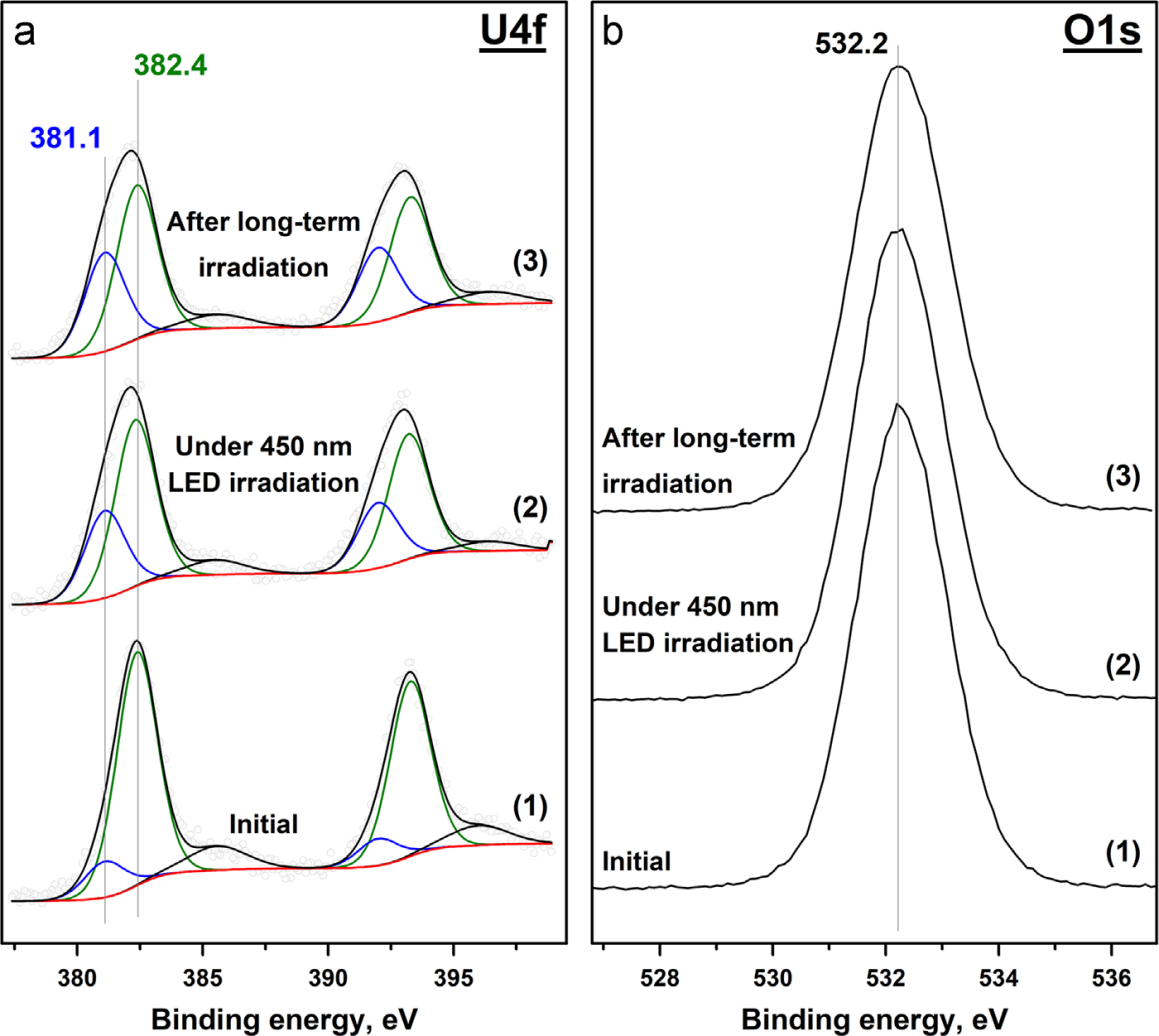
Photoelectron U4f (a) and O1s (b) spectral regions for the **5US** sample. The plots correspond to: the initial sample (1); after 450 nm LED irradiation for 60 min (2); the final state after long-term irradiation (3).

**Fig. 2:**
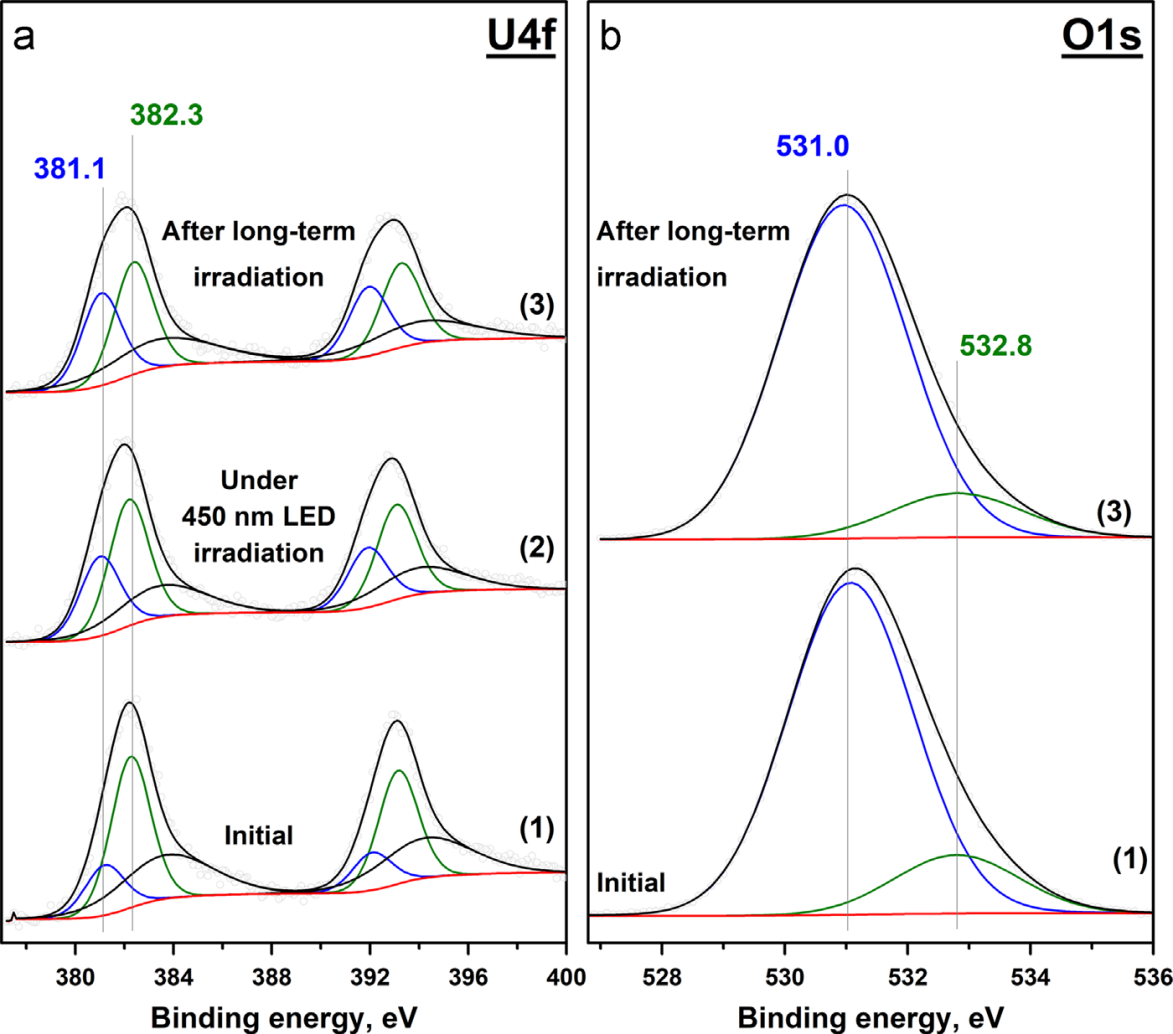
Photoelectron U4f (a) and O1s (b) spectral regions for the **5UA** sample. The plots correspond to: the initial sample (1); after 450 nm LED irradiation for 30 min (2); the final state after long-term irradiation.

**Fig. 3:**
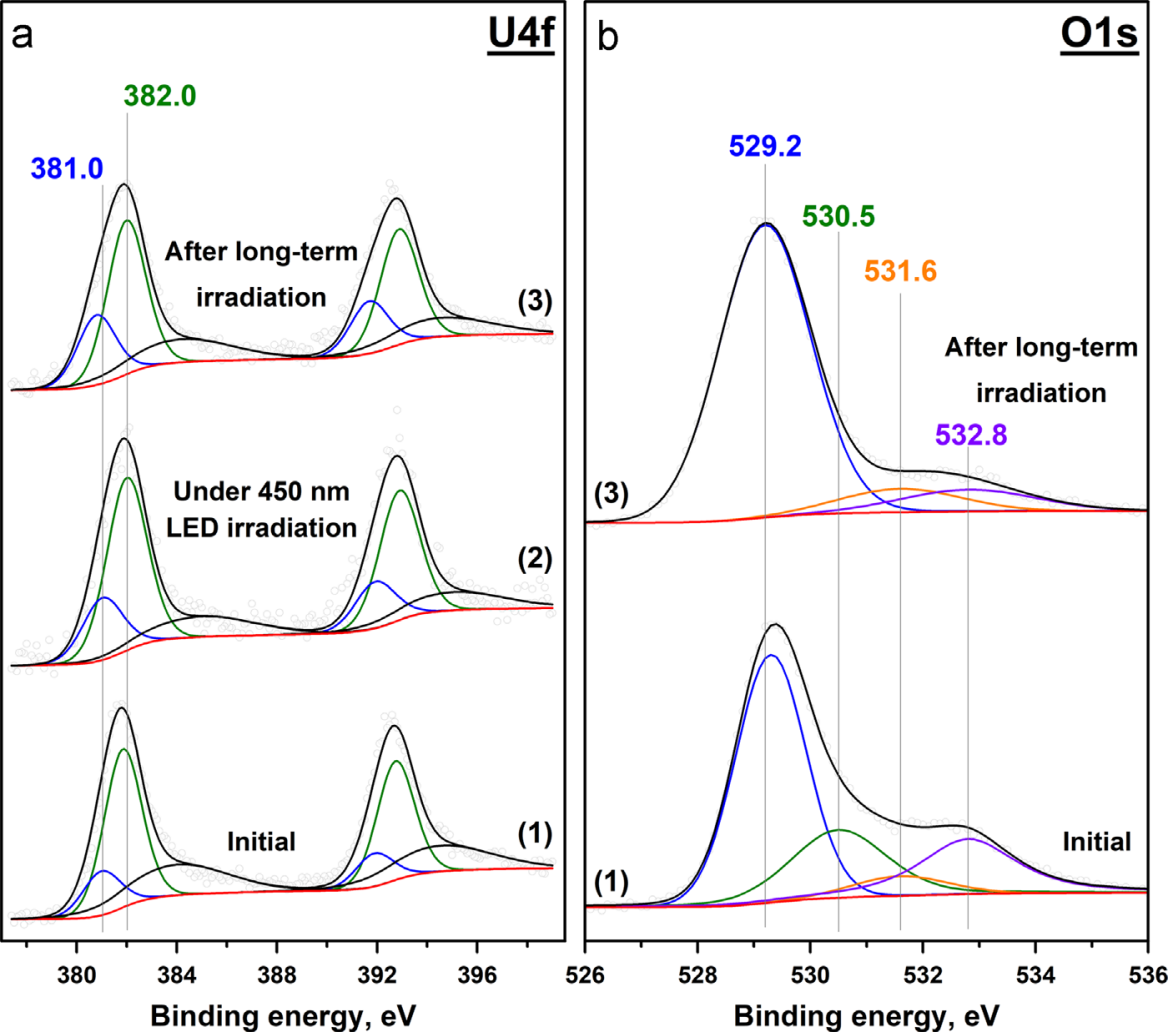
Photoelectron U4f (a) and O1s (b) spectral regions for the **5UC** sample. The plots correspond to: the initial sample (1); after 450 nm LED irradiation for 10 min (2); the final state after long-term irradiation (3).

**Fig. 4:**
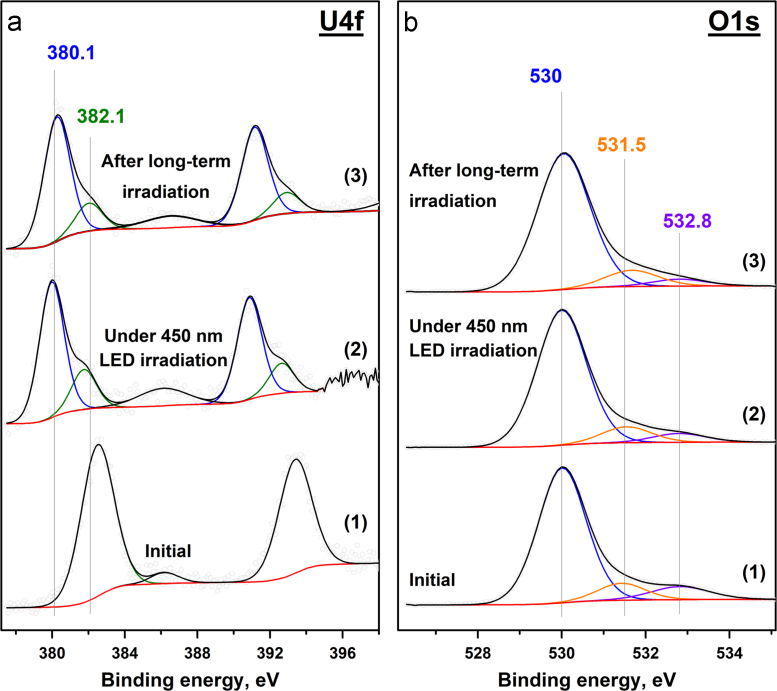
Photoelectron U4f (a) and O1s (b) spectral regions for the **5UT** sample. The plots correspond to: the initial sample (1); after 450 nm LED irradiation for 100 min (2); the final state after long-term irradiation (3).

In this study, *in situ* XPS experiments when the sample in a spectrometer chamber was simultaneously irradiated using 450 nm LED were performed to understand the behavior of uranyl species under the visible light on the surface of different oxides. Fig. 1, Fig. 2, Fig. 3, Fig. 4 show the photoelectron U4f and O1s spectral regions for the 5 wt% uranyl-loaded samples based on SiO_2_ (**5US**), Al_2_O_3_ (**5UA**), CeO_2_ (**5UC**), and TiO_2_ (**5UT**), respectively, before and after irradiation using 450 nm LED. According to literature data [1], [2], [3], [4], [5], [6], different positions of the U4f_7/2_ peaks (Fig. 1, Fig. 2, Fig. 3, Fig. 4a) can be attributed for different charge states of uranium. The relationship between the binding energy and corresponding charge state of uranium is shown in Table 1.

**Table 1:** Relationship between the position of U4f_7/2_ peak and the charge state of uranium.

Charge state	Binding energy (U4f_7/2_), eV
U^6+^	382.0–382.4
U^5+^	381.1
U^4+^	380.1

The peaks observed in the O1s spectral region (Fig. 1, Fig. 2, Fig. 3, Fig. 4b) can be attributed to the lattice oxygen, surface oxygen, oxygen from water and OH groups. The relationship between the position of the O1s peaks and the chemical state of oxygen was suggested on the basis of previously published papers [7], [8], [9]. The results for each sample are shown in Table 2.

**Table 2:** Relationship between the position of O1s peak and the chemical state of oxygen in the samples.

Chemical state of oxygen	Binding energy (O1s), eV			
	**5US**	**5UA**	**5UC**	**5UT**
Lattice (O_lat_)	532.2 eV	531.0 eV	529.2 eV	530.0 eV
Surface	–	–	530.5 eV	–
ОН	–	–	531.6 eV	531.5 eV
H_2_O	–	532.8 eV	532.8 eV	532.8 eV

The collected photoelectron spectra were used for the calculation of the ratio between the different chemical states of uranium and oxygen. The data for each sample before and after long-term 450 nm LED irradiation are shown in Table 3. In the table, lattice oxygen is referred to as O_lat_, and the combination of the other oxygen forms is marked as O_rest_.

**Table 3:** Ratio between the different chemical states of uranium and oxygen in the absence and presence of 450 nm LED irradiation.

	**5US**		**5UA**		**5UC**		**5UT**	
	U^5+^/U^6+^	O_rest_/O_lat_	U^5+^/U^6+^	O_rest_/O_lat_	U^5+^/U^6+^	O_rest_/O_lat_	U^4+^/U^6+^	O_rest_/O_lat_
Initial	0.15	0	0.33	0.17	0.28	0.92	0	0.24
Under irradiation	0.53	0	0.63	–	0.36	–	3.33	0.19
After long-term irradiation	0.63	0	0.83	0.14	0.45	0.21	5	0.17

## 2 Experimental design, materials, and methods

### 2.1 Materials

The uranyl nitrate hexahydrate (UO_2_(NO_3_)_2_ × 6H_2_O) from Izotop LLC (Russia) was employed for the modification of silica, alumina, ceria, and titania supports. SiO_2_ Type H (amorphous, *a*_s,BET_ = 440 m^2^/g) from Sigma-Aldrich (USA), Al_2_O_3_ (γ-phase, *a*_s,BET_ = 180 m^2^/g) from AO AZKiOS (Russia), CeO_2_ (fluorite-type, *a*_s,BET_ = 110 m^2^/g) prepared by the precipitation from a cerium nitrate solution with an aqueous ammonia solution [10], and TiO_2_ Hombifine N (100% anatase, *a*_s,BET_ = 350 m^2^/g) from Sachtleben Chemie GmbH (Germany) were used as supports.

### 2.2 Sample preparation

The silica, alumina, ceria, and titania supports were modified with uranyl ions via the impregnation method using UO_2_(NO_3_)_2_ aqueous solution [11]. Typically, 0.5 g of a support was suspended in 10 mL of deionized water, and 1 mL of 0.063 M aqueous solution of UO_2_(NO_3_)_2_ was added to the suspension. The theoretically calculated content of UO_2_(NO_3_)_2_ in the samples was 5 wt%. After vigorous stirring for 30 min, the suspension was evaporated. The precipitate was dried at 100 °C in air followed by the final grinding using an agate mortar and pestle.

### 2.3 X-ray photoelectron spectroscopy

**Fig. 5:**
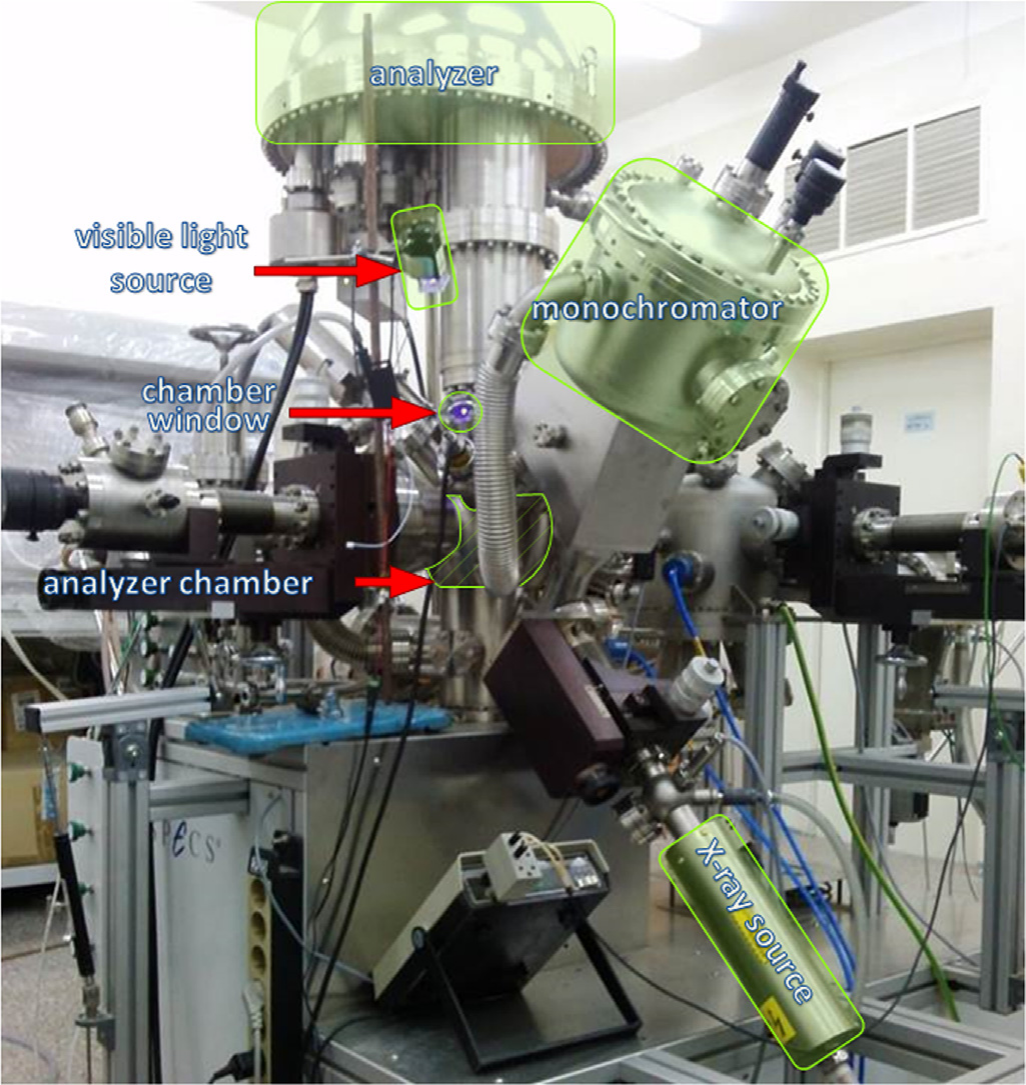
Photograph of the experimental setup used for *in situ* XPS measurements.

A SPECS (Germany) spectrometer equipped with a hemispherical PHOIBOS-150-MCD-9 analyzer and FOCUS-500 (AlKα radiation, *hλ* = 1486.74 eV, 200 W radiation power) monochromator was employed for *in situ* XPS experiments. In addition to a typical packaging, the presence of a special window in the analyzer chamber allowed for simultaneous irradiation of the investigated sample with visible light during the spectra collecting (Fig. 5). The sample preparation for the XPS analysis was performed under room irradiation from luminescent lamps. The position of the oxide-forming element for support was used as an internal standard for the calibration of photoelectron spectra. The analysis of each sample was performed as follows:

(1) Spectra collecting for the initial sample (*i.e*., without 450 nm LED irradiation);
(2) Spectra collecting for the sample 450 nm LED irradiated for a certain period;
(3) Spectra collecting after long-term irradiation (*i.e*., 1–2 h) and turning LED off.

The ratio between different charge states of a particular element was calculated using the areas of corresponding spectral lines or curve-fitted peaks, respectively.
