# Supplementary material for: In situ XPS data for the uranyl-modified oxides under visible light
Source: Data Brief. 2018 Jul 5;19:2053–60. doi: 10.1016/j.dib.2018.06.121 (PMC6141519; doi:10.1016/j.dib.2018.06.121)
Supplement: Supplementary file 1 — Transparency document [file mmc1.docx]

**Conflict of Interest Form**

We have no conflicts of interest to disclose.

Sincerely,

Selishchev Dmitry, PhD

Researcher Associate

Group of Photocatalysis

Boreskov Institute of Catalysis
